# Wide-awake local anaesthesia no tourniquet (WALANT) vs regional or general anaesthesia for flexor tendon repair in adults: protocol for a systematic review and meta-analysis

**DOI:** 10.1186/s13643-020-01532-1

**Published:** 2020-11-21

**Authors:** Grant S. Nolan, Ailbhe L. Kiely, Tomas Madura, Alexia Karantana

**Affiliations:** 1 Division of Surgery and Interventional Science, University College London, Royal Free Hospital, Pond Street, London, NW3 2QG UK; 2 Department of Plastic and Reconstructive Surgery, St Helens and Knowsley Teaching Hospitals NHS Trust, Whiston Hospital, Warrington Road, Prescot, Merseyside, L35 5DR UK; 3 Department of Plastic and Reconstructive Surgery, Queen Elizabeth Hospital, University Hospitals Birmingham NHS Trust, Mindelsohn Way, Edgbaston, B15 2TH UK; 4 Centre for Evidence Based Hand Surgery, School of Medicine, University of Nottingham, Derby Road, Nottingham, NG7 2UH UK

**Keywords:** Flexor tendon injury, Tendon injuries, Hand injuries, Finger injuries, Wide-awake, Anaesthetics, Local, WALANT, Wide-awake local anaesthesia no tourniquet, Adrenaline, Systematic review

## Abstract

**Background:**

Flexor tendon injuries most commonly occur following a penetrating injury to the hand or wrist. These are challenging injuries and the standard treatment is surgical repair under general or regional anaesthesia.

‘Wide-awake’ surgery is an emerging technique in hand surgery where a conscious patient is operated on under local anaesthetic. The vasoconstrictive effect of adrenaline (epinephrine) creates a ‘bloodless’ operating field and a tourniquet is not required. The potential advantages include intra-operative testing of the repair; removal of the risks of general anaesthesia; reduced costs; no aerosol generation from intubation therefore reduced risk of COVID-19 spread to healthcare professionals. The aim of this study will be to systematically evaluate the evidence to determine if wide-awake surgery is superior to general/regional anaesthetic in adults who undergo flexor tendon repair.

**Methods:**

We designed and registered a study protocol for a systematic review and meta-analysis of comparative and non-comparative studies. The primary outcome will be functional active range of motion. Secondary outcomes will be complications, resource use (operative time) and patient-reported outcome measures. A comprehensive literature search will be conducted (from 1946 to present) in MEDLINE, EMBASE, CINAHL, and Cochrane Library. Grey literature will be identified through Open Grey, dissertation databases and clinical trials registers. All studies on wide-awake surgery for flexor tendon repair will be included. The comparator will be general or regional anaesthesia. No limitations will be imposed on peer review status or language of publication. Two investigators will independently screen all citations, full-text articles and abstract data. Potential conflicts will be resolved through discussion or referral to a third author when necessary. The study methodological quality (or bias) will be appraised using an appropriate tool. If feasible, we will conduct a random effects meta-analysis.

**Discussion:**

This systematic review will summarise the best available evidence and definitively establish if function, complications, cost, or patient-reported outcomes are improved when flexor tendons are repaired using wide-awake technique. It will determine if this novel approach is superior to general or regional anaesthesia. This knowledge will help guide hand surgeons by continuing to improve outcomes from flexor tendon injuries.

**Systematic review registration:**

PROSPERO CRD42020182196

**Supplementary Information:**

The online version contains supplementary material available at 10.1186/s13643-020-01532-1.

## Introduction

Flexor tendon injuries result in the inability to bend the fingers or thumb and occur following an injury to flexor digitorum profundus (FDP), flexor digitorum superficialis (FDS) or flexor policies longus (FPL) tendons. This is most commonly due to a penetrating injury which sharply divides the tendon which is located superficially in the hand or wrist. The incidence of flexor tendon injuries in the United Kingdom (UK) is estimated at 41/100,000 person years [1, 2]. These injuries disproportionately affect males of working age [2] and have the potential to cause significant morbidity due to poor functional outcomes which affect livelihoods. In the UK, they are managed exclusively in specialist plastic surgery or orthopaedic hand centres due to the challenging nature of these injuries for surgeons and patients alike.

Treatment of flexor tendon injuries includes surgical repair, where the two ends are retrieved and sutured together. This can be made more difficult by a delay in time to surgery, as the unopposed flexor muscles cause the cut tendons to retract proximally [3]. Sometimes it is necessary to dissect into the palm or the carpal tunnel for retrieval. Historically, a tourniquet has been used to create a bloodless field. As flexor tendon repair can have a prolonged operative time, the tourniquet makes a general anaesthetic (GA) unavoidable, as tourniquets become intolerable to a conscious patient after approximately 20 min [4].

An emerging technique in hand surgery is a ‘wide-awake’ approach, where a conscious patient is operated on under local anaesthesia [5, 6]. The bloodless field is achieved without a tourniquet by harnessing the vasoconstrictive effect of adrenaline (epinephrine), which is injected with the local anaesthetic. Adrenaline in fingers is safe [7] and the myth that it causes digital necrosis has been thoroughly debunked through a multi-centre prospective study of over 3000 patients undergoing injections into fingers with adrenaline [7, 8]. This approach has been coined “wide-awake local anaesthesia no tourniquet” or WALANT by a Canadian hand surgeon [6].

There are many potential advantages of WALANT for patients and surgeons alike [6]. With an awake patient, the repair can be tested for gapping and impingement on pulleys, which can be remedied immediately to reduce complications of rupture and adhesions. WALANT overcomes the inconveniences and risks of GA, allowing the infirm and high-risk patient to undergo flexor tendon repair, overcoming the need for fasting and facilitating same-day discharge. An awake patient allows the delivery of crucial patient education throughout the surgery through dialogue with the operating surgeon [9]. Costs may be reduced through reduced staff (no anaesthetist), reduces consumables and clinic requirements, as the patient has been fully educated throughout the procedure [9]. Of relevance to the current health climate, performing the procedure under local anaesthetic also removes aerosol generation due to intubation and extubating, so reduces the risk to healthcare professionals from COVID-19. Whilst the technique has only been around for a decade, a 2020 survey of American hand surgeons showed that uptake was modest, with only 10% using WALANT for flexor tendon repair [10].

The aim of this study will be to systematically evaluate the evidence to determine the efficacy of WALANT compared to other anaesthetic techniques with a tourniquet in terms of functional outcomes, complications, costs and patient-reported outcomes for adults who undergo flexor tendon repair.

## Methods

This protocol has been registered with PROSPERO international prospective register of systematic reviews (registration number CRD42020182196) and has been reported in accordance with the Preferred Reporting Items for Systematic Reviews and Meta-Analysis Protocols (PRISMA-P) 2015 statement [11]. The PRISMA-P checklist for this study is included in Additional file 1. The methodology of this review will be according to the Cochrane Handbook for Systematic Review of Interventions [12]. The final review will be reported following the PRISMA statement and the Meta-Analysis of Observational Studies in Epidemiology (MOOSE) guidelines [13].

### Information sources and search strategy

The primary source of literature will be a structured search of the following major electronic databases from 1946 to May 2020: MEDLINE, EMBASE, CINAHL and Cochrane Library.

The secondary source of potentially relevant material will be a search of the grey or difficult to locate literature including OpenGrey, Dissertation databases (e.g. Open Access Thesis and Dissertations) and Clinical trial registers (e.g. ClinicalTrials.gov). We will perform hand searching of the reference lists of included studies, relevant reviews, national clinical practice guidelines or other relevant documents to identify cited articles not captured by electronic searches. Content experts and authors who are prolific in the field will be contacted. The literature searches will be designed and conducted by the review team in conjunction with an information specialist. The search will be performed in English. Translations will be obtained for non-English articles. The search will include a broad range of terms and keywords related to flexor tendon injuries and wide-awake surgery. No limitations will be imposed on peer review status or language of publication. A draft search strategy for MEDLINE is provided in Additional file 2.

### Study selection

Study selection will be conducted in a two-stage process. The titles, and if required the abstracts, will initially be screened by two reviewers, using pre-specified screening criteria, for potential eligibility after excluding duplicate records. This process will be performed in Rayyan [14], which is a bespoke web and mobile app for systematic reviews. Relevant studies will then undergo full-text review where available by both reviewers. Translations will be obtained for non-English articles. Any discrepancies between reviewers will be resolved by discussion or referral to a third author. The search results, including abstracts, full-text articles and record of the reviewer’s decisions, including reasons for exclusion, will be recorded in a combination of Rayyan [14] and Microsoft Excel (Microsoft Corporation, 2018).

### Eligibility criteria

Studies will be selected according to the following criteria: participants, condition or outcome of interest and study design. No limitations will be imposed on peer review status or language of publication.

### Participants/population

We will include studies involving adult patients (i.e. ≥ 16 years old at time of surgery) who undergo primary surgical repair of flexor tendons using WALANT. In studies with mixed populations, we will include the study if > 90% of participants fulfils the entry criteria for the systematic review or if results for eligible participants are reported separately (i.e. subgroups).

Flexor tendon injuries (diagnostic code S66.0/1) occur when either the FDP, FDS or FPL tendons are damaged in the hand or wrist.

Patients who suffered amputations or replants, closed tendon ruptures, secondary tendon reconstruction or segmental tendon loss will be excluded.

### Intervention

We will include all studies that report outcomes from primary flexor tendon repair in adults using wide-awake surgery. Wide-awake local anaesthesia no tourniquet (WALANT) or wide-awake surgery is the use of local anaesthetic agents such as lidocaine in combination with adrenaline (epinephrine) applied directly to the area of surgery. The local anaesthesia is injected into the subcutaneous tissues in a tumescent fashion over the area of interest. This eliminates pain and the vasoconstrictive effect of adrenaline reduces bleeding and provides a relatively bloodless operating field.

### Comparator

The comparator will be other anaesthetic techniques such as GA or regional anaesthesia (i.e. block). Unless specified, it is assumed that these techniques are combined with a tourniquet to create a bloodless field.

### Outcome measures

The primary outcome measures will be functional active range of motion, reported categorically as ‘good’ or ‘excellent’ outcomes, as measured by a validated tool (adjusted Strickland score [15], total active motion [TAM] score) [16]. Results from two different range of motion scores will not be combined unless scores can be re-calculated from raw data requested from the study authors. Secondary outcomes will be complications (e.g. rupture, adhesions requiring tenolysis, infection, failure of WALANT requiring conversion to GA, etc.), resource use (operative time) and patient-reported outcome measures including pain. Both primary and secondary outcomes will be reported at short (less than 3 months), medium (between 3 and 12 months) and long-term (more than 12 months).

### Study design

Eligible studies will be both interventional and observational in nature. All comparative studies which report outcomes from patients undergoing primary flexor tendon repair via WALANT and GA or regional anaesthesia will be included. Non-comparative studies solely reporting outcomes from WALANT for primary flexor tendon repair will also be included. Studies solely reporting outcomes from primary tendon repair under GA or regional anaesthetic will be excluded. We will exclude letters, reviews, case reports and case series with fewer than three flexor tendon injuries.

### Setting

Studies performed in any setting will be included.

### Data extraction

The data from all articles included in the review will be independently retrieved by two reviewers using a standardised electronic data extraction form in Microsoft Excel (Microsoft Corporation, 2018).

- Study characteristics.
  - ○ Authors, year of publication, journal, country, study design, language of publication.
  - ○ Inclusion criteria, exclusion criteria.
- Time period of data collection
- Type of control anaesthesia (GA/regional/combination of GA and regional)
- Patient demographics
  - ○ Total number of patients
  - ○ Number excluded
  - ○ Number of males/females
  - ○ Mean/median age + standard deviation/interquartile range
  - ○ Method of epitendinous and core suture repair (including type of suture used)
  - ○ Information of venting of pulleys
  - ○ Rehabilitation protocol used
- Number of tendons injuries per group (WALANT, GA/regional)
- Primary outcomes
  - ○ Functional active range of motion (adjusted Strickland or TAM score)
- Secondary outcomes
  - ○ Complications
  - ○ Resource use
  - ○ Patient-reported outcome measures

In the case of missing data, we will contact authors via email asking them to provide these details. As we do not expect authors of studies published more than 10 years ago to respond to inquiries, we will only contact authors of studies published from 2010 onwards. Research has previously shown about 30% of trial authors is unreachable, and 40% do not respond to emails, even after several reminders [17]. Therefore, if no reply is forthcoming or the message cannot be delivered, then we will not try to contact the authors again.

For continuous outcomes, we will impute the standard deviation from *p* values according to the guidance in the Cochrane Handbook for Systematic Reviews of Interventions [12]. If the data are likely to be normally distributed, we will use the median for meta-analysis when the mean is not available. If it is not possible to calculate the standard deviation from the *p* value or the confidence intervals, we will impute the standard deviation using the largest standard deviation in other studies for that outcome. This form of imputation can decrease the weight of the study for calculation of mean differences and may bias the effect estimate to no effect for calculation of standardised mean differences [12].

### Assessment of risk of bias of included studies

The risk of bias will be assessed at the individual study level by two review authors independently. Discrepancies will be resolved through discussion or referral to a third author if required. The included studies will be interventional and observational. Some studies may be uncontrolled (e.g. case series on WALANT for flexor tendon repairs.) Each of these will be assessed using a relevant tool to their study design.

Randomised trials will be assessed using the Cochrane Risk of Bias tool [18], non-randomised comparative studies (e.g. cohort and case-control studies) will be assessed using ROBINS-I tool [19], uncontrolled studies (e.g. case series) will be assessed using a tool specifically developed for these type of studies [20], which is formed from an adaptation of previous criteria from Pierson [21], Bradford Hills [22] and Newcastle Ottawa scale [23].

### Data analysis and synthesis

The data from included studies will initially be used to build evidence tables. This will include study characteristics, context, participants, outcomes and findings. If the raw data is available for studies which report range of motion, then this will be used to calculate an adjusted Strickland score [24] which will be used preferentially due to its specificity to the action of a flexor tendon. The adjusted Strickland scores are then converted into ‘excellent’, ‘good’, ‘fair’, or ‘poor’ scores in the standard manner [24]. We will calculate the relative risk of achieving a ‘good’ or ‘excellent’ functional outcome and the relative risk of achieving a ‘fair’ or ‘poor’ functional outcome. These will each be presented with their 95% confidence intervals. Where range of motion cannot be combined, we will only combine those who report functional ROM using similar scores, i.e. studies reporting TAM scores with other similar studies.

For the secondary outcomes, the relative risk of each type of complication (e.g. tendon rupture, adhesions requiring tenolysis, conversion of WALANT to general anaesthesia etc.) will also be presented with 95% confidence intervals. For resource use, we will calculate the mean difference in operative time with 95% confidence intervals. We will calculate the mean difference or standardised mean difference with 95% confidence intervals for patient-reported outcome measures (depending on if multiple studies have used the same or different assessment scales).

If only non-comparative studies are identified in the systematic review, then a crude incidence estimate of ‘good’ or ‘excellent’ ROM, and each complication (number of events/sample size) will be calculated by meta-analysis of proportion and presented along with 95% confidence intervals. The results of these will be presented in forest plots.

To determine the extent of variation between selected studies, separate tests of heterogeneity will be performed for randomised trials and non-randomised comparative studies. Inter-study heterogeneity will be assessed visually using the forest plot. Statistical heterogeneity will be quantified statistically using three tests. The *I*^2^ statistic will be used and the result will be interpreted using the definitions in the Cochrane Handbook for Systematic Reviews of Interventions [12]. If two or more comparative studies, with an absence of clinical heterogeneity, are identified by the systematic review then we will perform a random-effects meta-analysis. If two or more non-comparative studies are identified with a lack of clinical heterogeneity (e.g. in terms of method of tendon repair and rehab protocol) and then we will perform a meta-analysis of proportions with a random-effects model [25]. This approach is appropriate given it is likely that the true value varies from study to study and these follow a normal distribution.

If two or more comparative studies are identified by this systematic review then a summary of findings table will be created for the primary outcome measure. We will rate the overall quality of evidence of these outcomes using the Grading of Recommendations, Assessment, Development and Evaluation (GRADE) Working Group methodology [26].

### Additional analysis

We will undertake a sensitivity analysis to ensure the robustness of our results. The results of the risk of bias tools will be used in a sensitivity analysis to ensure studies judged to be at ‘high’ risk of bias do not affect the robustness of our results in any subsequent meta-analysis.

Firstly, studies judged to be at high risk of bias will be excluded and secondly excluding those studies in which any values were imputed. If contributing studies have sufficient data, subgroup analysis will be performed on outcomes for zone II flexor tendon injuries.

### Meta-biases

Small study effects (or publication bias across studies) will be assessed and a funnel plot will be generated for each meta-analysis containing 10 or more studies. Publication bias will be assessed by inspecting a funnel plot for asymmetry and with Egger’s test [27] where appropriate, with the results considered to indicate potential small study effects when *p* values are < 0.10, if more than 10 studies are included.

## Discussion

Despite many advances in surgery, flexor tendon injuries remain a challenge to both surgeons and patients alike. Poor functional outcomes affect people of working age, which adds to the morbidity of these injuries. Wide-awake surgery is an emerging technique which arguably has many benefits over the standard approach of using GA/regional anaesthesia and a tourniquet [6]. This systematic review will aim to summarise the best available evidence and definitively establish which approach is superior in terms of function, reduced complications, cost and improved patient-reported outcomes. This knowledge will help guide hand surgeons by continuing to improve outcomes in flexor tendon injuries.

In this paper, we have presented a study protocol for a systematic review with meta-analysis. If any amendments or deviations from the protocol are required, these will be reported in the final manuscript. We plan to disseminate the results of this systematic review at national meetings/conferences of hand surgeons (both orthopaedic and plastic surgeons) and through publication.

At the study level, there are several limitations that we anticipate. As this is an emerging technique there may be a paucity of evidence on the topic, and trials may not have yet finished recruitment or published their results. Additionally, there are other patients and surgical factors which may affect the incidence of complications, (e.g. diabetes or grade of operating surgeon affecting infection and rupture rate respectively) but these factors are infrequently reported in studies. In non-randomised studies, these factors can be confounders.

Finally, we expect a degree of reporting bias (through both non-publication of studies and selective reporting of outcomes) which may affect this systematic review. As with any surgical intervention, there is always a potential conflict of interest in terms of reporting one’s own treatment results. We hope to minimise this through contacting authors for missing details and identifying publication bias using a funnel plot.

## Supplementary Information

**Additional file 1.** PRISMA-P 2015 Checklist.

**Additional file 2.** Search strategy for MEDLINE (Ovid).

## Data Availability

Not applicable.

## Abbreviations

CINAHL: The Cumulative Index to Nursing and Allied Health Literature
COVID-19: Coronavirus disease 2019
EMBASE: Excerpta Medica Database
FDP: Flexor digitorum profundus tendon
FDS: Flexor digitorum superficialis tendon
FPL: Flexor policies longus tendon
GA: General anaesthetic
GRADE: Grade of Recommendations, Assessment, Development and Evaluation
MeSH: Medical Subject Headings
MOOSE: Meta-Analysis of Observational Studies in Epidemiology
NHS: National Health Service
PRISMA-P: Preferred Reporting Items for Systematic Review and Meta-Analysis Protocols
RCTs: Randomised controlled trials
RoB 2: Cochrane Risk of Bias 2 tool
ROBINS-I: Risk Of Bias In Non-randomised Studies - of Interventions
WALANT: Wide-awake local anaesthesia no tourniquet
UK: United Kingdom of Great Britain and Northern Ireland
