# Supplementary material for: Wide-awake local anaesthesia no tourniquet (WALANT) vs regional or general anaesthesia for flexor tendon repair in adults: protocol for a systematic review and meta-analysis
Source: Syst Rev. 2020 Nov 21;9:264. doi: 10.1186/s13643-020-01532-1 (PMC7680064; doi:10.1186/s13643-020-01532-1)
Supplement: Supplementary file 2 — Additional file 2. Search strategy for MEDLINE (Ovid). [file 13643_2020_1532_MOESM2_ESM.docx]

Search strategy for MEDLINE (Ovid)

1. Tendon Injuries/
2. Flexor.mp. [mp=title, abstract, original title, name of substance word, subject heading word, floating sub-heading word, keyword heading word, organism supplementary concept word, protocol supplementary concept word, rare disease supplementary concept word, unique identifier, synonyms]
3. Tendon.mp. [mp=title, abstract, original title, name of substance word, subject heading word, floating sub-heading word, keyword heading word, organism supplementary concept word, protocol supplementary concept word, rare disease supplementary concept word, unique identifier, synonyms]
4. Wide awake.mp. [mp=title, abstract, original title, name of substance word, subject heading word, floating sub-heading word, keyword heading word, organism supplementary concept word, protocol supplementary concept word, rare disease supplementary concept word, unique identifier, synonyms]
5. Wide-awake.mp. [mp=title, abstract, original title, name of substance word, subject heading word, floating sub-heading word, keyword heading word, organism supplementary concept word, protocol supplementary concept word, rare disease supplementary concept word, unique identifier, synonyms]
6. Anesthetics, Local/
7. Anesthesia, Local/
8. Lidocaine/
9. Bupivacaine/
10. WALANT.mp. [mp=title, abstract, original title, name of substance word, subject heading word, floating sub-heading word, keyword heading word, organism supplementary concept word, protocol supplementary concept word, rare disease supplementary concept word, unique identifier, synonyms]
11. local anaest*.mp. [mp=title, abstract, original title, name of substance word, subject heading word, floating sub-heading word, keyword heading word, organism supplementary concept word, protocol supplementary concept word, rare disease supplementary concept word, unique identifier, synonyms]
12. 1 or 2 or 3
13. 4 or 5 or 6 or 7 or 8 or 9 or 10 or 11
14. 12 and 13
